# Supplementary material for: Sugars dominate the seagrass rhizosphere
Source: Nat Ecol Evol. 2022 May 2;6(7):866–77. doi: 10.1038/s41559-022-01740-z (PMC9262712; doi:10.1038/s41559-022-01740-z)
Supplement: Supplementary file 1 — Supplementary Text 1–4, Methods and Figs. 1–12. [file 41559_2022_1740_MOESM1_ESM.pdf]

nature portfolio

https://doi.org/10.1038/s41559-022-01740-z

Supplementary information

Sugars dominate the seagrass rhizosphere

In the format provided by the authors and unedited

Sugars dominate the seagrass rhizosphere

Author List:
E. Maggie Sogin[1,2*]; Dolma Michellod[1]; Harald R. Gruber-Vodicka[1]; Patric Bourceau[1,3]; Benedikt Geier[1]; Dimitri V. Meier[4]; Michael Seidel[5]; Soeren Ahmerkamp[1]; Sina Schorn[1]; Grace D'Angelo[1], Gabriele Procaccini[6]; Nicole Dubilier[1,3*]; Manuel Liebeke[1*]

Affiliations:
1- Max Planck Institute for Marine Microbiology, Bremen, Germany
2- University of California, Merced, CA, USA
3- MARUM—Center for Marine Environmental Sciences of the University of Bremen, Bremen, Germany
4- Institute of Biogeochemistry and Pollutant Dynamics, Department of Environmental Systems Science, Swiss Federal Institute of Technology, Zurich
5- Institute for Chemistry and Biology of the Marine Environment, University of Oldenburg, Oldenburg, Germany
6- Stazione Zoologica Anton Dohrn, Napoli, Italy

* Corresponding Authors:

E. Maggie Sogin; esogin@ucmerced.edu
Nicole Dubilier; ndubilie@mpi-bremen.de
Manuel Liebeke; mliebeke@mpi-bremen.de

Supplementary Information

Supplementary Text 1

Temporal cycling of sugars. Sugar concentrations in Mediterranean *P. oceanica* tissues and sediments were correlated to the availability of light on both seasonal and daily time scales. Our seasonal analyses revealed significantly higher sucrose concentrations in sediment pore waters underneath a *P. oceanica* meadow in April than in October or July (**Figure S3; Table S2** and **Table S3**). These observations are consistent with *P. oceanica's* growing season over the summer months. The observed decline in sucrose concentrations in July, along with previous observations of decreases over the summer in total organic carbon and bulk carbohydrates in sediments underneath *P. oceanica*[1-3], suggest that the seagrass diverted these organic carbon resources to support growth during their main productivity season. Also, sediment microbial communities could be more active during the summer months when temperatures are higher[4], leading to increased removal of sucrose.

To understand daily fluctuations of sucrose, we measured sucrose concentrations at discrete sampling time points over 24 hours in late spring in both *P. oceanica* tissues and their sediments. Our data indicates that during the day, *P. oceanica* produces sucrose in its leaves as a product of photosynthesis, which is transferred to the roots and rhizomes where it is released into the pore water. Sucrose abundances in *P. oceanica* leaves, but not their rhizomes or roots, were significantly higher at dusk than at all other sampling time points (**Extended Data 3a-d; Table S2** and **Table S3**). Changes in sucrose abundances within *P. oceanica* leaves thus

followed the diel ramping up and down of photosynthetic and respiration rates[5-7]. Relatively stable sucrose concentrations in *P. oceanica*'s roots and rhizomes over 24 hours suggest that the regulation of sucrose in these organs is independent of diel rhythms. Within the pore waters underneath the seagrass, sucrose concentrations were significantly higher during the day than at night (**Extended Data 3e; Table S2** and **Table S3**). In summary, our data indicates excess sucrose production during peak productivity hours is transferred to the underlying sediments, where it is only partly consumed during the night.

Zostera marina. We observed less then 2 µM of sucrose (**Table S1**) underneath *Z. marina* meadows in the Baltic sea, which is three orders of magnitude lower than the maximum sucrose concentrations observed underneath the Mediterranean and Caribbean seagrass meadows investigated in this study (**Table S1**). There are likely many environmental and biological factors that can explain our observations. Further studies are needed to determine factors that govern the limited sucrose accumulation underneath *Z. marina* meadows.

Supplementary Text 2

Sediment incubations. Sediment slurry incubations can lead to higher metabolic rates than in situ sediment core incubations, which typically experience lower rates of substrate diffusion[8-10]. However, as we showed, sucrose concentrations are consistently high in the *P. oceanica's* rhizosphere, suggesting that in situ, sediment sugar concentrations are not limited by diffusion. Therefore, we choose to use slurry incubations to mimic in situ conditions and ensure the sediment community had constant access to labeled sucrose and exposure to phenolic compounds.

To examine if seagrass-derived phenolics inhibit microbial respiration of sucrose, we incubated sediments from a Mediterranean *P. oceanica* meadow with labeled $^{13}C_{12}$-sucrose in the presence and absence of oxygen as well as seagrass-derived phenolics. While sediment slurry experiments can increase the rates of microbial metabolism in comparison to similar studies performed with intact cores[8-10], we choose to use bottle incubations to more accurately manipulate the sediment conditions in order to identify a mechanism limiting sucrose removal. The volumetric rates determined during these incubations were converted to areal rates assuming an active layer of 3 cm for oxic conditions and 20 cm under anoxia. Under oxic conditions, sediments collected in the upper 3 cm depth showed similar removal rates of sucrose across all treatments at 86 mmol(C)/m²d^{-1}. The rates of $^{13}CO_2$ released corresponded well with removal rates and ranged between 64 and 83 mmol(C)/m²d^{-1} (**Table S5**). These experimental rates are unlikely to be significant in situ since the concentration of sucrose close to the sediment surface is low at an average of 0.35 µM. Under oxic conditions, we assume the phenolics were quickly oxidized and removed from the incubation, thereby allowing the microbial community from inside the meadow to respire sucrose (**Figure 4**).

Sediment collected from the anoxic layers underneath the meadow and diluted 15-fold with anoxic seawater, removed sucrose at a rate corresponding to 1891 mmol(C)/m²d^{-1}, and respired sucrose fully to CO_2 at a rate of 232 mmol(C)/m²d^{-1}. Flushing the sediment with five volumes of artificial, anoxic seawater before the incubation (to remove seagrass-derived phenolics), reduced the rates slightly to 1589 mmol(C)/m²d^{-1} and 158 mmol(C)/m²d^{-1}. Lower metabolic rates in the flushed sediments could also be related to the removal of microbial cells or nutrients during flushing. Since we consistently detected high concentrations of sucrose under the meadows, there must be another inhibitory effect in the environment. Finally, when we added the phenolic extract from the seagrass tissues to the incubations conducted in artificial seawater, we overserved a significantly lower rate of sucrose removal 169.21 mmol (C)/m²d^{-1} and $^{13}CO_2$ production at 20 mm(C)/m^{2}d^{-1}. While it is possible that we may have introduced other metabolites to these incubations in trace amounts during the addition of the phenolic compounds, we are confident that the sediment microbial community did not use these

compounds in their metabolism. This is because the background $^{12}CO_2$ values obtained during the IRMS data acquisition revealed no change in microbial respiration between artificial seawater treatments (**Figure S12**).

Supplementary Text 3

Metabolic rate calculations. To calculate metabolic rates from sediment incubation experiments, we prepared a standard curve for different concentrations of carbonate with the natural abundance of ^{12}C and ^{13}C in artificial seawater. We used this curve to determine the concentration of $^{13}CO_2$ and $^{12}CO_2$ from individual samples as described in the methods. The value for $^{13}CO_2$ was corrected for the background $^{13}CO_2$ based on the natural abundance of carbon. The amount of $^{13}CO_2$ produced was calculated by multiplying the concentration of $^{13}CO_2$ in the sample by the incubation volume. This was divided by the sediment volume and the change in time to determine a rate of ^{13}C production in mol ml(sediment)$^{-1}$ h^{-1}. The areal rates were extrapolated from these incubation experiments by multiplying this rate by the volume of the sucrose rich layer based on our field observations in per m^2.

Supplementary Text 4

Expression of sucrose orthologs. While metatranscriptomics is not definitive proof of in situ activity, gene expression data is commonly accepted as a proxy for the microbial use of a given substrate[11-13]. Using our metagenomically assembled genomes (MAGs) from the seagrass sediments, we mapped transcriptomic reads to determine the relative expression of individual orthologs across the genome bin for each habitat. To analyze if sucrose degradation was central to the metabolism of the recovered MAGs, we compared the proportion of transcripts for sucrose degradation to all expressed orthologs in transcripts per million base pairs (TPM) within a MAG. The expression of sucrose degradation genes in most MAGs, with a few exceptions, were not among the top 10% of all expressed orthologs at all sampling sites, including those directly underneath the meadow with the highest sucrose concentrations (**Figure S9**).

General metabolism of putative sucrose specialists. To determine the metabolism of the six putative sucrose specialists, we looked for the expression of key marker genes involved in select metabolic pathways. Because our MAGs were incomplete (72.8% to 94.8%), it is possible some metabolic genes are missing from the MAGs.

Both MAGs 76 and 154 lacked a cytochrome c terminal oxidase, making them likely anaerobes as the MAGs do not have the genes needed for oxygen respiration. MAG 154 (*Desulfosarcinaceae sp.)* contained genes that encoded for sulfate reduction (dissimilatory sulfite reductase; EC 1.8.99.3), hydrogen oxidation (hydrogenase large and small subunit; EC 1.12.99.6), and nitrogen fixation (including the nitrogenase proteins: *NifA*, *NifB*, *NifE*, and *NifH*), all of which were expressed. MAG 154 was more abundant inside the meadow then at the edge or outside (**Figure S10**). We predict this population of bacteria is capable of fermenting plant-based sugars based on the expression patterns of multiple types of glycoside hydrolases. MAG 76 (*Beggiatoales sp*.) contained genes that encoded for sulfur oxidation (*SoxB*, *SoxX*, *SoxY*, and *SoxZ*) and a gene that encoded for a protein that uses nitrate as an electron acceptor (respiratory nitrate reductase, EC 1.7.99.4). It also had genes that encoded for hydrogen oxidation (hydrogenase large and small subunit; EC 1.12.99.6) and nitrogen fixation (nitrogenase beta and alpha chains; EC 1.18.6.1). These genes were also expressed in our sediment metatranscriptomes.

The remaining four MAGs contained genes for oxygen respiration which were expressed in the transcriptomic libraries. Expression data indicated MAG 207 (*Thiohalomonadales sp.)* fixed CO_2 through the Calvin Cycle (Ribulose bisphosphate carboxylase, EC 4.1.1.39), oxidized

reduced sulfur compounds (*SoxA, SoxB, SoxY, SoxZ*) and possibly hydrogen (hydrogenase, EC 1.12.99.6), reduced nitrite to nitrous oxide (nitric-oxide reductase, EC 1.7.99.7) and fixes nitrogen (nitrogenase; *NifA, NifB*; nitric-oxide reductase EC 1.7.99.7). MAG 142 (Xanthomonadales) also contained genes that encoded for the autotrophic fixation of CO_2 through the Calvin cycle (EC 4.1.1.39) and hydrogen oxidation (hydrogenase, EC 1.12.99.6), both of which were expressed in the sediment metatranscriptomes. MAG 209 (*Verrucomicrobiales*) and 438 (*Gammaproteobacteria sp.*) contained genes for the general uptake of organic material through heterotrophy.

Five out of the six MAGs also contained genes involved in the breakdown of phenolics, many of which were expressed the sediment metatranscriptomes. This is intriguing since many phenolic compounds limit bacterial growth and produce significant antibacterial activity, often accompanied by an increase of reactive oxygen species[14]. Some bacteria respond to the presence of phenolics with the production of antioxidants, like glutathione or enzymes like catalase and dismutase to counterbalance reactive oxygen stress. The putative sucrose specialists in our study have at least two pathways for defense against phenolics: They expressed genes for phenolic degradation pathways (benzoyl-CoA and beta-ketoadipate pathways) and the production of antioxidant compounds and enzymes (e.g. catalase, superoxide dismutase, and glutathione peroxidase). Our data suggest the expression of these enzymes enables these taxa to use sucrose, and other sugars, in their metabolism (**Table S8**).

Supplementary Methods

Pore water sampling. We designed a porewater lance to facilitate sampling of seagrass sediments. The tip was rounded to allow the lance to penetrate the sediment without piercing the root tissues. The blunt steel lance was 1 m long, 2 µm inner diameter outfitted with a wire mesh (63 µm) to prevent the intake of sediment and peat material, pore water was slowly extracted from sediments into sterile syringes. As evidence that the pore water lance did not piece the root tissues, the *P. oceanica* root metabolome, as detected by GC-MS, was significantly different then the porewater itself (**Figure S11**). In this case, using steel lances was preferable because the sediments at our sampling sties were quite coarse, and typical coring approaches that were developed for fine-grained sediments would lose a large amount of porewater during sampling. Furthermore, lances were essential for a fine-scale extraction of sediment porewaters and have been used by others to study the in situ chemistry of seagrass meadows[15,16], as well as for optode-based oxygen measurements in saltmarshes[17].

A pore water profile consisted of top to bottom sampling of the sediments every 5 or 10 cm down to 30 or 40 cm depending on sampling location. Samples were frozen at -20°C until processing for metabolomic analyses. Pore water profiles were collected at higher spatial and temporal scales from two *P. oceanica* meadows off the island of Elba, Italy. Profiles (n=3) from replicate (n=3) transects were collected inside, 1 m at the edge and 20 m outside a meadow in Sant'Andrea Bay, Italy (42° 48'29.4588" N; 10° 8' 34.4436" E ; 6-8 m water depth). These collections were repeated in April, July, and October of 2016 to describe metabolite composition across *P. oceanica's* growing season.

To explore how pore water metabolite composition changes across a diurnal cycle, pore water samples were collected at 10 cm sediment depth during a 24 hour diurnal cycle in May 2017 in Galanzana Bay, Elba, Italy (42° 44'9.438" N; 10° 14' 16.3032" E; approximately 2 m water depth). Using replicate lances fixed in the sediment throughout the experiment (n=10) sediment water was extracted (2 mL) at time points around high and low solar irradiance periods (10:00, 12:00, 14:00, 20:00, 22:00, 0:00, 2:00 and 5:00) on 23-24, May 2017. Following collection, samples were immersed in liquid nitrogen and subsequently stored at -20 °C until further processing.

Seagrass sampling and extraction for metabolomics. Individual *P. oceanica* plants from Galanzana Bay were sampled during a diurnal cycle (at 10:00, 12:00, 14:00, 20:00, 0:00, and 5:00) from the same meadow and in parallel with sediment pore water samples. Individual plants were collected at least 1 m away from the fixed pore water lances to avoid impacting variation in sediment chemistry. Immediately following collection (< 5 min), whole plants were immersed in liquid nitrogen to halt changes in metabolite composition. Plants were stored at -80 °C until metabolites extraction.

Metabolites were extracted from seagrass tissues using a modified method for plant-based metabolite profiling[18]. Briefly, tissues from frozen plants were separated into leaves, rhizomes, and roots and ground under liquid nitrogen to a fine powder using mortar and pestle. Approximately 400 mg of ground plant tissue was aliquoted into Eppendorf vials. Pre-cooled methanol (1.4 mL) was added to each aliquot and vortexed for 10 s. Tissues were extracted at 70 °C for 10 min in a thermomixer at 950 rpm and subsequently centrifuged to pellet the tissue at 11,000 *g* at 4 °C. The supernatant was transferred to 14 mL glass vials. To separate the polar and non-polar compounds, 750 µL of chloroform and 1.5 mL of high-grade water was added to each sample, vortexed for 10 s and centrifuged for 15 min at 2,200 *g*. 25 µL of the upper, polar phase was dried to completeness in a vacuum concentrator without heating (approximately 1 h) after which the aliquots were stored at -80 °C until metabolite derivatization (as described in the **Methods** for pore water samples).

GC-MS data processing using XCMS. GC-MS profiles were processed using XCMS (v2.99.6)[19] in R. Individual peaks were picked using the matchedFilter algorithm with a full width at half maximum set to 8.4, signal to noise threshold at 1, *m*/*z* width of 0.25 (step parameter), and *m*/*z* difference between overlapping peaks at 1. Resulting peaks were grouped, retention times corrected and regrouped using the density (bandwidth parameter set to 2) and obiwarp methods. Following peak filling, the CAMERA (v1.32.0)[20] package was used to place *m/z* peaks into pseudo-spectra by grouping similar peaks with the groupFWHM function. A single *m/z* value was selected to represent each CAMERA group using the following criteria: 1) *m/z* value > 150, 2) occurs across samples with the highest frequency and, if two or more ions are tied in terms of number of samples detected, 3) has the highest mean intensity. Ions with very low mean intensities (< 0.001) were considered noise and removed from the analysis.

Preparation of pore water samples for DOM analysis. Briefly, Agilent Bond Elut PPL (0.1 g) cartridges were conditioned with methanol followed by ultrapure water (pH 2 using 25% hydrochloric acid). 15 to 20 mL of acidified pore water (pH 2) was loaded onto the cartridge followed by another rinse with ultrapure water (pH 2). The cartridges were dried with argon gas and DOM was eluted with 0.5 mL methanol and stored at −20 °C until further analysis. The extraction efficiencies were determined as DOC in methanol extract divided by bulk DOC concentrations of the original pore water samples. DOC in methanol extracts was determined by drying an aliquot of extract and re-dissolving in ultrapure water. The extraction efficiency was 37 ± 11%. Methanol extracts with isolated DOM were diluted in 1:1 methanol/water to a final concentration of 5 mg C L^{-1}. The samples were analyzed by ultrahigh-resolution mass spectrometry as previously described[21] using a solariX XR Fourier transform ion cyclotron resonance mass spectrometer (FT-ICR-MS, Bruker Daltonik GmbH, Bremen, Germany) connected to a 15 T superconducting magnet. The samples were analyzed in negative mode and infused into the electrospray source (Apollo II ion source, Bruker Daltonik GmbH, Bremen, Germany) at 2 µL min^{-1}. Two hundred scans were collected with a mass window from 92 to 2000 Da. Molecular formulae above the method detection limit[22] were assigned with the following restrictions: $^{12}C_{1-30}$ $^{1}H_{1-200}$ O_{1-50} $^{14}N_{0-4}$ S_{0-2} P_{0-1}. Molecular masses were removed for further analysis when detected in less than three samples. Samples were normalized to the sum

of FT-ICR-MS signal intensities and a modified aromaticity index (AI_{mod})[23] was calculated for each sample. Molecular formulae were identified as polyphenolic and/or highly aromatic compounds if $0.66 \geq AI_{mod} \geq 0.50$[21].

Sample preparation for mass spectrometry imaging. Individual seagrass root tissue sections were obtained by pressing the 12 µm thick tissue sample between two frozen Polysine glass slides to ensure a flat surface for mass spectrometry imaging (MSI) analysis. Before MSI sample preparation, overview images of the root sections were acquired with a stereomicroscope (SMZ25, Nikon, Düsseedorf, Germany) and, for the high-resolution figure in the manuscript, with an Olympus BX53 compound microscope (Olympus, Tokyo, Japan) at a resolution of 0.36 µm/ pixel, operated through the software cellSens (Olympus, Tokyo, Japan). For matrix application, super 2,5-dihydroxybenzoic acid solution (S-DBH; 30 mg mL^{-1} in 50% methanol:water with 0.1% trifluoroacetic acid (TFA) was applied to each section using an automated pneumatic sprayer (SunCollect, SunChrome, Wissenschaftliche Geräte GmbH, Germany). Spraying consisted of 10 layers at a flow rate of 10 µL min^{-1} (layer 1) and 15 µL min^{-1} (layers 2-10) resulting in a homogeneous layer of crystalline matrix with crystal sizes around 1 µm. To identify phenolic compounds within seagrass root tissues, an additional seagrass root section was prepared by applying 9-aminoacridine matrix (9AA) in 70% methanol:water with 0.1% TFA using an automated pneumatic sprayer. Spraying consisted of 8 layers in total at a flow rate of 10 µL min^{-1} (layer 1), 20 µL min^{-1} (layer 2), 30 µL min^{-1} (layer 3) and 40 µL min^{-1} (layers 4-8).

Oxygen detection instrument. To measure oxygen concentrations in situ, an oxygen flow-through cell (Pyroscience, OXFTC) was connected to a stainless steel push-point sampling lance. The inside of the oxygen flow-through cell is coated with an oxygen-sensitive fluorescent dye, which allows contactless oxygen measurements from outside of the cell. Dye excitation and emission readout was performed via a custom-made glass fiber connected to an underwater oxygen OEM module (Pyroscience, FSO2-SUBPORT). The oxygen module data was stored using an analog logger from a diver-operated motorized microsensor profiler system (DOMS, see[24]). Prior to sampling, the oxygen flow-through cell was calibrated using a two-point calibration procedure. First, aerated sea water was percolated through the chamber (100%) followed by sea water treated with natrium dithionate (0%). Importantly, our method did not provide sufficient resolution to detect microscale gradients in subsurface peaks in oxygen close to seagrass roots, as shown in previous studies[25].

Preparation of phenolic extract from seagrass tissues. A crude extract was prepared from *P. oceanica* tissues by extracting 18.2 g of wet plant material in methanol:water (vol./vol., 50:50) for 24 hours. The tissue was subsequently re-extracted in fresh solvent for a second 24 hour period and the resulting extracts were combined. The phenolic compounds were purified by evaporating the extract to dryness, resuspension in water and defatting with chloroform. The aqueous solution was extracted three times with ethyl acetate and then three times with n-butanol. The phenolic fraction was obtained from the n-butanol phase, which was evaporated to dryness under vacuum in a rotary evaporator[26].

LC-MS analysis of SPE pore water extracts. LC-MS/MS was performed using a ThermoFisher Vanquish uHPLC instrument. Analytes were separated on a C18 column (Accucore Vanquish C18+; 100 mm x 2.1 mm; LOT 14906)), with a flow rate of 0.200 ml/min at 40 °C using the following LC gradient consisting of water (A) and methanol in 0.1 % formic acid (B): 2 min 100 % A, a linear increase over 16 min to 95 % B followed by 6 min at 95 % B, a linear decrease to 5 % B followed by 2 min at 5 % B. UV traces were measured at 275 nm, 325

nm, 210 nm, and 3DField. All samples and standards were measured in negative mode with a scan range of 150 -1200 m/z, 70000 mass resolution, and with top 5 MS/MS fragmentations. For identification of caffeic acid, a pure chemical standard solution was prepared and analyzed using the LC-MS method described above.

DNA extraction for metagenomics. Extracellular DNA (exDNA) was removed by suspending frozen sediments into 200 µL of carbonate dissolution buffer. The solution was incubated at room temperature for 1 h at 600 rpm, 1.6 mL of 10X tris hydrochloride buffer (300 mM; pH 10) added to each sample, and incubated for an additional hour. All samples were centrifuged at 10,000 x g for 20 min to separate dissolved exDNA from sediments containing intact microbial cells. Remaining cellular DNA was extracted from pelleted sediments in 500 µL of extraction buffer[27]. Following 3 freeze thaw cycles, 10 µL of proteinase K (20 mg μL^{-1}) was added to each sample and incubated for 30 min at 37 °C. Each sample was then incubated (2 h, 50 °C) with 50 µL of 20% SDS buffer and then centrifuged at 4000 x *g* for 10 min. After re-extracting the pellet in both buffers (166 µL of extraction buffer and 16 µL of 20% SDS at 65 °C for 20 min), sample supernatants were combined with equal volumes of chloroform: isoamylalcohol (24:1 v/v) and centrifuged (10,000 x *g*, 10 min). This step was repeated a second time and the resulting aqueous phase containing DNA was combined with 600 µL of isopropanol and stored overnight at 4 °C. To pellet DNA, samples were centrifuged (14,000 x *g*, 30 min) and the supernatant discarded. The DNA pellet was washed in pre-cooled 80% ethanol and centrifuged (14,000 x *g*, 10 min). The resulting supernatant was removed and the DNA pellet was dried in a vacuum concentrator (Eppendorf concentrator plus, 10 min, V-AL). DNA was eluted in DEPC water and stored at -20 °C until metagenomic library preparation.

RNA extraction for metatranscriptomics. RNA from sediment samples was extracted as follows: 0.2 g of each sediment sample was homogenized in 500 µL of lysis solution I (30 mM Tris-HCL, 30 mM EDTA, 800 mM guanidium hydrochloride, 0.5% TritonX-100) and flash frozen in liquid nitrogen. Samples were thawed at 65 °C under gentle agitation, and then vortexed for 10 s and incubated for 60 min at 50 °C under constant shaking. The resulting mixture was flash frozen in liquid nitrogen and thawed at 65 °C, after which 500 µL of lysis solution II (2.5 M NaCl, 2% CTAB, 0.1% PVPP) was added to the mixture. Following two more freeze-thaw cycles, samples were incubated for 60 min at 65 °C. Samples were centrifuged for 30 min 5242 x *g* at 4 °C and resulting supernatant transferred for purification. Supernatants containing the nucleic acids were purified using two cycles of washing in 1 volume of chloroform:isoamylalcohol (24:1), inverting 60 times, and centrifuging the mixture for 30 min at 5242 x *g* at 4 °C. The nucleic acids were precipitated from the resulting aqueous supernatant using the following steps: fully dissolve linear polyacrylamide was added to each sample to achieve a final concentration of 20 $\mu L\ mL^{-1}$, samples were homogenize with 0.1 volumes of 5 M sodium chloride solution following by 1.5 volumes of isopropanol, this mixture was incubated in the dark at -20 °C overnight, and centrifuged for 30 min at 14,000 x *g* at 4 °C. The nucleic acids were washed by adding 70% ethanol and centrifuged for 10 min at 14,000 x *g* at 4 °C. The pellet was air dried and dissolved in 100 µL of RNAase free water. Following this purification step, the resulting RNA was further cleaned using the Norgen Biotec CallAll kit (BioCat # 23800-NB) following manufacturer's protocol.

Library preparation and sequencing. To construct metagenomic sequencing libraries, 10 ng of genomic DNA was fragmented via sonication (Covaris S2, Covaris) followed by library preparation with NEBNext Ultra II FS DNA Library Prep Kit for Illumina (New England Biolabs). DNA library preparation included 6 cycles of PCR amplification. Total RNA was used for cDNA generation Ovation RNA-Seq system V2 (NuGEN) according to the manufacturer's specifications. Subsequently, 500 ng cDNA was fragmented via sonication, followed by library

preparation with NEBNext Ultra II FS DNA Library Prep Kit for Illumina. Library preparation included 5 cycles of PCR amplification. Quality and quantity were assessed at all steps for all libraries via capillary electrophoresis (TapeStation, Agilent Technologies) and fluorometry (Qubit, Thermo Fisher Scientific). All libraries were immobilized and processed onto a flow cell with cBot (Illumina) and subsequently sequenced on HiSeq3000 system (Illumina) with 2 x 150 bp paired end reads.

To obtain 16S rRNA reads, amplicon sequencing libraries were constructed for PacBio sequencing of the 16S rRNA gene. Briefly, the sediment DNA was purified with DNeasy PowerClean Pro Cleanup Kit, Qiagen. 16S amplicons were amplified with the universal primer pair GM3F (5′-AGAGTTTGATCMTGGC-3′) /GM4R (5′-TACCTTGTTACGACTT-3′)[28] and Phusion DNA polymerase, following the New England Biolabs routine PCR protocol including the addition of DMSO with annealing temperature for the first two cycles at 42 °C, 20 s followed by 45 °C, 20 s for the remaining 34 cycles. The number of PCR cycles for sufficient DNA for downstream applications was empirically determined by quantitative PCR (ViiA 7 Real-Time PCR System, ThermoFisher) in the presence of 1X Evagreen (20X Evagreen, Biotium, Fremont, USA). Amplicons were purified with DNA Clean & Concentrator Kits, Zymo Research or AMpure Beads (Beckman Coulter) and quantified by Qubit HS kit (ThermoFisher). Next, PacBio libraries were prepared as recommended in protocol "2 kb Template Preparation and Sequencing" of Pacific Biosciences using adaptors with 16mer barcode sequence to demultiplex sequencing data. Libraries were pooled equimolar and sequenced on Sequel I of Pacific Biosciences on a single cell with 20 hours run time, polymerase binding kit 3.0, sequencing chemistry version 3.0 and SMRT cell 1Mv3. Resulting reads were error corrected using circular consensus (CCS) reads (10 passes) using the SMRTlink (v 6.0.0) software.

References

1 Pirc, H. Growth dynamics in Posidonia oceanica (L.) Delile: I. seasonal changes of soluble carbohydrates, starch, free amino acids, nitrogen and organic anions in different parts of the plant. *Marine Ecology* **6**, 141-165 (1985).

2 Danovaro, R. Detritus-bacteria-meiofauna interactions in a seagrass bed (Posidonia oceanica) of the NW Mediterranean. *Marine Biology* **127**, 1-13 (1996).

3 Pirc, H. Seasonal aspects of photosynthesis in Posidonia oceanica: influence of depth, temperature and light intensity. *Aquatic Botany* **26**, 203-212 (1986).

4 Weston, N. B. & Joye, S. B. Temperature-driven decoupling of key phases of organic matter degradation in marine sediments. *Proceedings of the National Academy of Sciences* **102**, 17036-17040 (2005).

5 Koopmans, D., Holtappels, M., Chennu, A., Weber, M. & de Beer, D. High Net Primary Production of Mediterranean Seagrass (Posidonia oceanica) Meadows Determined With Aquatic Eddy Covariance. *Frontiers in Marine Science* **7**, doi:10.3389/fmars.2020.00118 (2020).

6 Champenois, W. & Borges, A. Seasonal and interannual variations of community metabolism rates of a Posidonia oceanica seagrass meadow. *Limnology and Oceanography* **57**, 347-361 (2012).

7 Procaccini, G. *et al.* Depth-specific fluctuations of gene expression and protein abundance modulate the photophysiology in the seagrass Posidonia oceanica. *Scientific reports* **7**, 1-15 (2017).

8 Kaspar, H. F. Denitrification in marine sediment: measurement of capacity and estimate of in situ rate. *Applied and Environmental Microbiology* **43**, 522-527 (1982).
9 Laverman, A. M., Van Cappellen, P., van Rotterdam-Los, D., Pallud, C. & Abell, J. Potential rates and pathways of microbial nitrate reduction in coastal sediments. *FEMS microbiology ecology* **58**, 179-192 (2006).
10 Pallud, C. & Van Cappellen, P. Kinetics of microbial sulfate reduction in estuarine sediments. *Geochimica et Cosmochimica Acta* **70**, 1148-1162 (2006).
11 Edlund, A. *et al.* Uncovering complex microbiome activities via metatranscriptomics during 24 hours of oral biofilm assembly and maturation. *Microbiome* **6**, 1-22 (2018).
12 Jones, D. S., Flood, B. E. & Bailey, J. V. Metatranscriptomic insights into polyphosphate metabolism in marine sediments. *The ISME journal* **10**, 1015-1019 (2016).
13 Broman, E., Sjöstedt, J., Pinhassi, J. & Dopson, M. Shifts in coastal sediment oxygenation cause pronounced changes in microbial community composition and associated metabolism. *Microbiome* **5**, 1-18 (2017).
14 Kohanski, M. A., Dwyer, D. J., Hayete, B., Lawrence, C. A. & Collins, J. J. A Common Mechanism of Cellular Death Induced by Bactericidal Antibiotics. *Cell* **130**, 797-810, doi:10.1016/j.cell.2007.06.049 (2007).
15 Ruff, S. E. *et al.* Methane Seep in Shallow-Water Permeable Sediment Harbors High Diversity of Anaerobic Methanotrophic Communities, Elba, Italy. *Frontiers in Microbiology* **7**, doi:10.3389/fmicb.2016.00374 (2016).
16 Mohr, W. *et al.* Terrestrial-type nitrogen-fixing symbiosis between seagrass and a marine bacterium. *Nature* **600**, 105-109 (2021).
17 Koop-Jakobsen, K., Fischer, J. & Wenzhöfer, F. Survey of sediment oxygenation in rhizospheres of the saltmarsh grass - Spartina anglica. *Science of The Total Environment* **589**, 191-199, doi:https://doi.org/10.1016/j.scitotenv.2017.02.147 (2017).
18 Lisec, J., Schauer, N., Kopka, J., Willmitzer, L. & Fernie, A. R. Gas chromatography mass spectrometry–based metabolite profiling in plants. *Nature protocols* **1**, 387-396 (2006).
19 Smith, C. A., Want, E. J., O'Maille, G., Abagyan, R. & Siuzdak, G. XCMS: processing mass spectrometry data for metabolite profiling using nonlinear peak alignment, matching, and identification. *Analytical chemistry* **78**, 779-787 (2006).
20 Kuhl, C., Tautenhahn, R., Bottcher, C., Larson, T. R. & Neumann, S. CAMERA: an integrated strategy for compound spectra extraction and annotation of liquid chromatography/mass spectrometry data sets. *Analytical chemistry* **84**, 283-289 (2012).
21 Seidel, M. *et al.* Biogeochemistry of dissolved organic matter in an anoxic intertidal creek bank. *Geochimica et Cosmochimica Acta* **140**, 418-434 (2014).
22 Riedel, T. & Dittmar, T. A method detection limit for the analysis of natural organic matter via Fourier transform ion cyclotron resonance mass spectrometry. *Analytical chemistry* **86**, 8376-8382 (2014).
23 Koch, B. P. & Dittmar, T. From mass to structure: An aromaticity index for high-resolution mass data of natural organic matter. *Rapid communications in mass spectrometry* **30**, 250-250 (2016).
24 Weber, M. *et al.* In situ applications of a new diver-operated motorized microsensor profiler. *Environmental science & technology* **41**, 6210-6215 (2007).

25 Martin, B. C. *et al.* Oxygen loss from seagrass roots coincides with colonisation of sulphide-oxidising cable bacteria and reduces sulphide stress. *The ISME journal* **13**, 707-719 (2019).

26 Grignon-Dubois, M. & Rezzonico, B. Phenolic fingerprint of the seagrass Posidonia oceanica from four locations in the Mediterranean Sea: first evidence for the large predominance of chicoric acid. *Botanica Marina* **58**, 379-391, doi:doi:10.1515/bot-2014-0098 (2015).

27 Zhou, J., Bruns, M. A. & Tiedje, J. M. DNA recovery from soils of diverse composition. *Applied and environmental microbiology* **62**, 316-322 (1996).

28 Ansede, J. H., Friedman, R. & Yoch, D. C. Phylogenetic analysis of culturable dimethyl sulfide-producing bacteria from a Spartina-dominated salt marsh and estuarine water. *Applied and Environmental Microbiology* **67**, 1210-1217 (2001).

29 Olivier, D., Costa, J., Desjobert, J.-M. & Pergent, G. Variations in the concentration of phenolic compounds in the seagrass Posidonia oceanica under conditions of competition. *Phytochemistry* **65**, 3211-3220, doi:https://doi.org/10.1016/j.phytochem.2004.09.003 (2004).

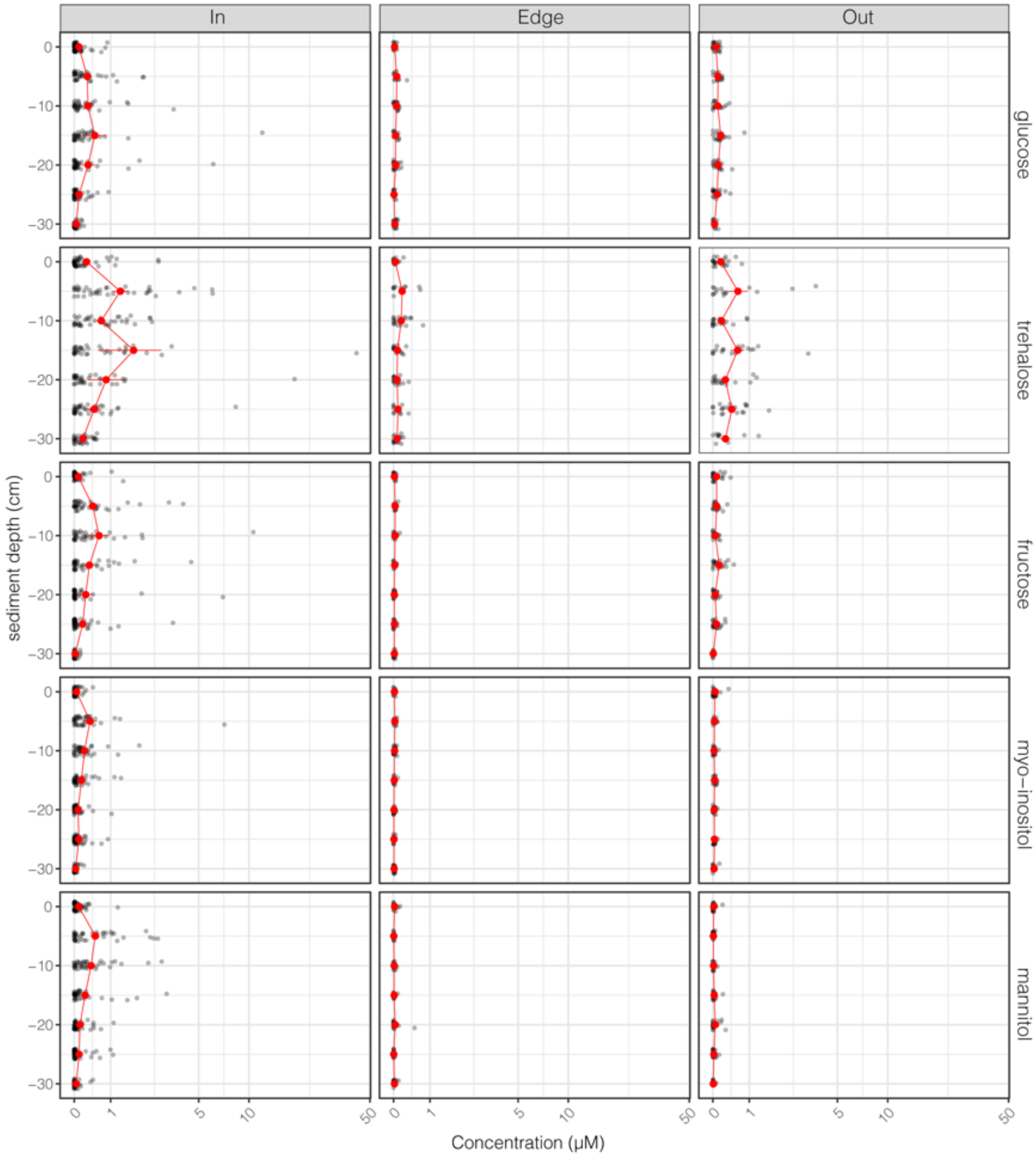

Supplementary Figure 1 | Simple sugars are more abundant inside *a P. oceanica* meadow than out. Plots show sugar concentrations in porewaters across sediment depth. Red points represent mean concentrations and error bars are standard error of the mean. Grey points represent individual peak values from replicate metabolomic profiles (Inside: 0 cm n=46, -5 cm n=40, -10 cm n=38, -15 cm n=38, -20 cm n=37, -25 cm n=42, -30 cm n=34; Edge: 0 cm n=14, -5 cm n=11, -10 cm n=17, -15 cm n=12, -20 cm n=18, -25 cm n=11, -30 cm n=16; Outside: 0 cm n=22, -5 cm n=15, -10 cm n=16, -15 cm n=18, -20 cm n=17, -25 cm n=19, -30 cm n=10), Two-way ANOVA results revealed that glucose (p = 0.0008), fructose (p = 0.047), myo-inositol (p = 0.000752), and mannitol (p < 0.00001) concentrations were significantly higher inside the meadow than outside or at the edge. While trehalose was not significantly different between sampling locations (p=0.21), it was significantly different across sampling depths (p=0.00069). All two-way ANOVA comparing sugar concentrations as a function of sediment depth and location, model results and significance values are reported in **Table S2** and **Table S3**.

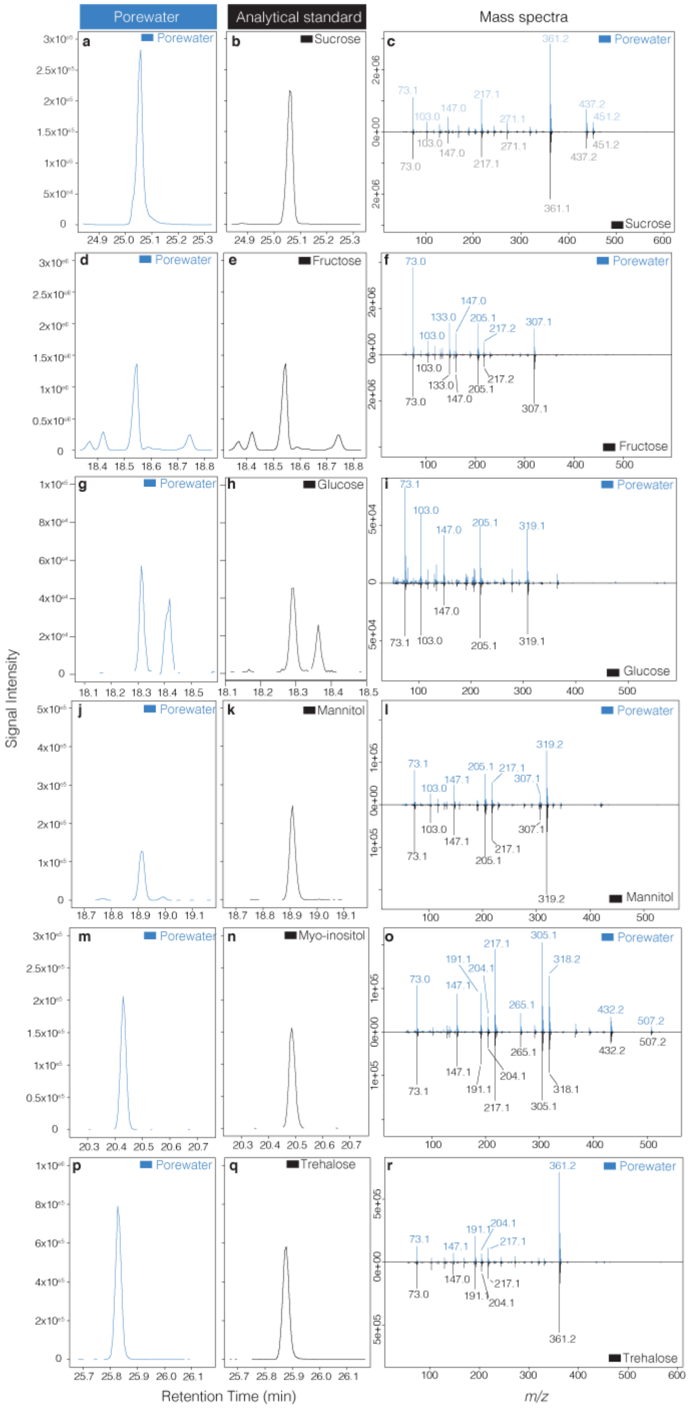

Supplementary Figure 2 | Identification of sugars from sediment porewaters. Sugars in sediment porewaters (**a, d, g, j, m,** and **p**) were identified by to the NIST database library and confirmed through comparison of both retention times (**b, e, h, k, n**, and **q**) and tail-to-tail mass spectra (**c, f, i, l, o,** and **r**) to an authentic standards. Total ion chromatograms and tail-to-tail mass spectra comparing the fragmentation pattern are shown for porewater peaks and authentic standards for sucrose (**a-c**), fructose (**d-f**), glucose (**g-i**), mannitol (**j-l**), myo-inositol (**m-o**), and trehalose (**p-r**).

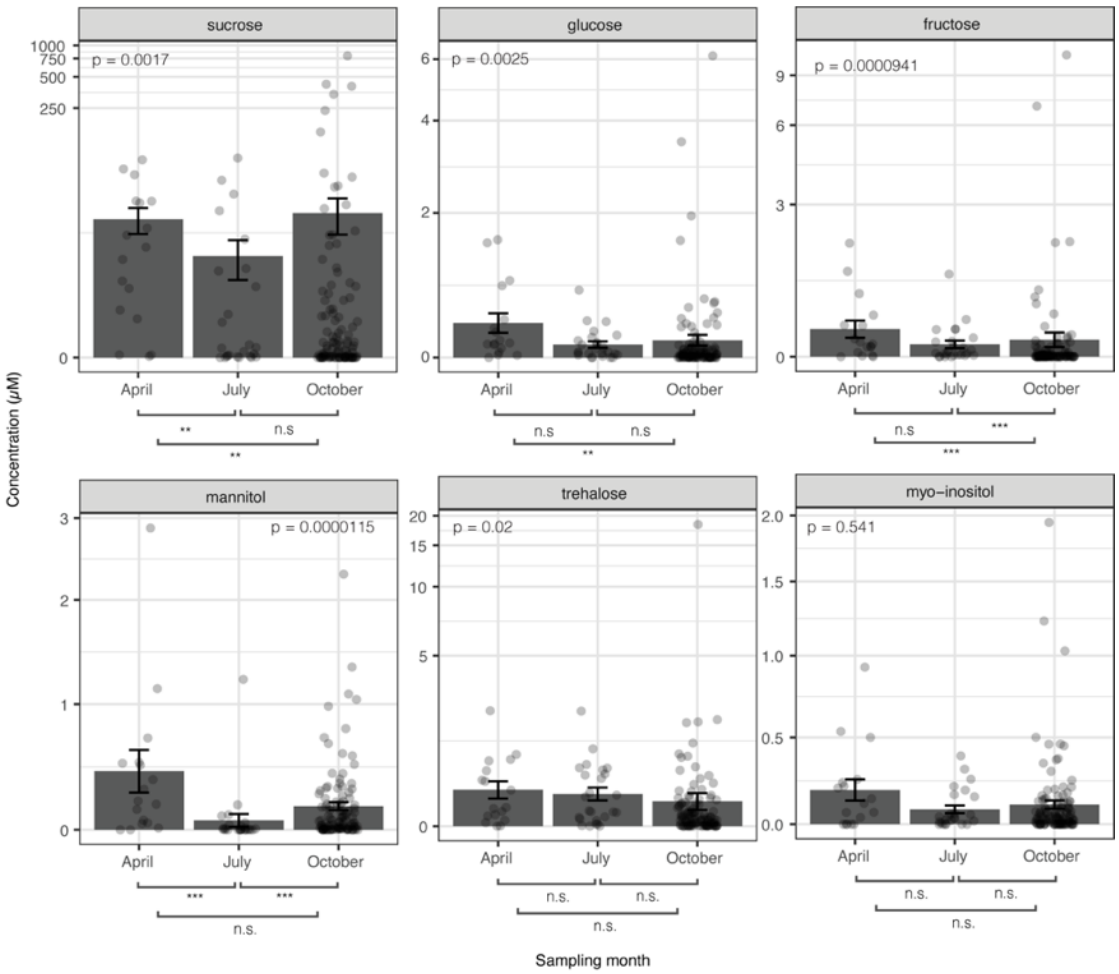

Supplementary Figure 3 | Sugar concentrations in the porewater underneath a *P. oceanica* meadow changed across seasons. Bar plots show average sugar concentrations for sucrose, glucose, fructose, and mannitol varied with sampling season. Post-hoc test reveal differences between sampling months following a two-way ANOVA testing sampling depth by sampling month; ****p-value* < 0.001, ** *p-value* < 0.01, n.s.= not significant; Bar plots show the mean concentration +/- SEM of the mean of each sugar for April (n=17), July (n=25) and October (n=113). Overlying points represent individual measurements (statistical results reported in **Table S2** and **Table S3).**

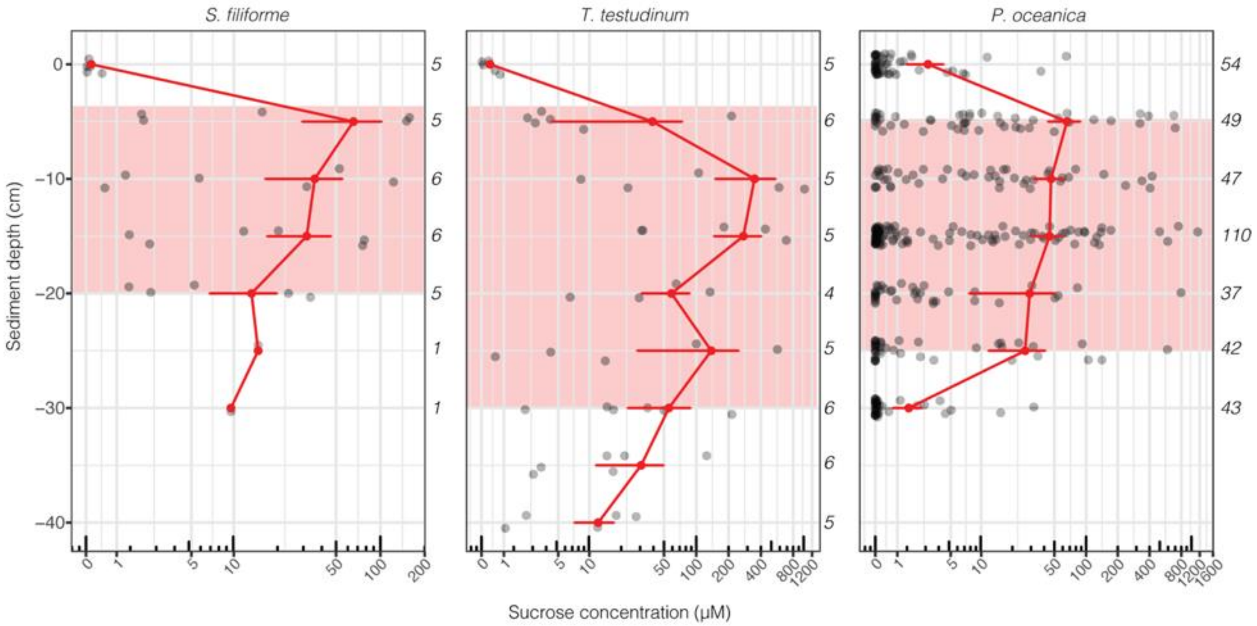

Supplementary Figure 4 | Porewater sucrose concentrations are highest at the sediment root interface. Red points and connected lines represent mean +/- SEM of sucrose concentrations (µM)(gray points; **Table S1**). Sample sizes are indicated in each figure panel across depths. Pink boxes show known depth of seagrass roots for each species based on literature reports.

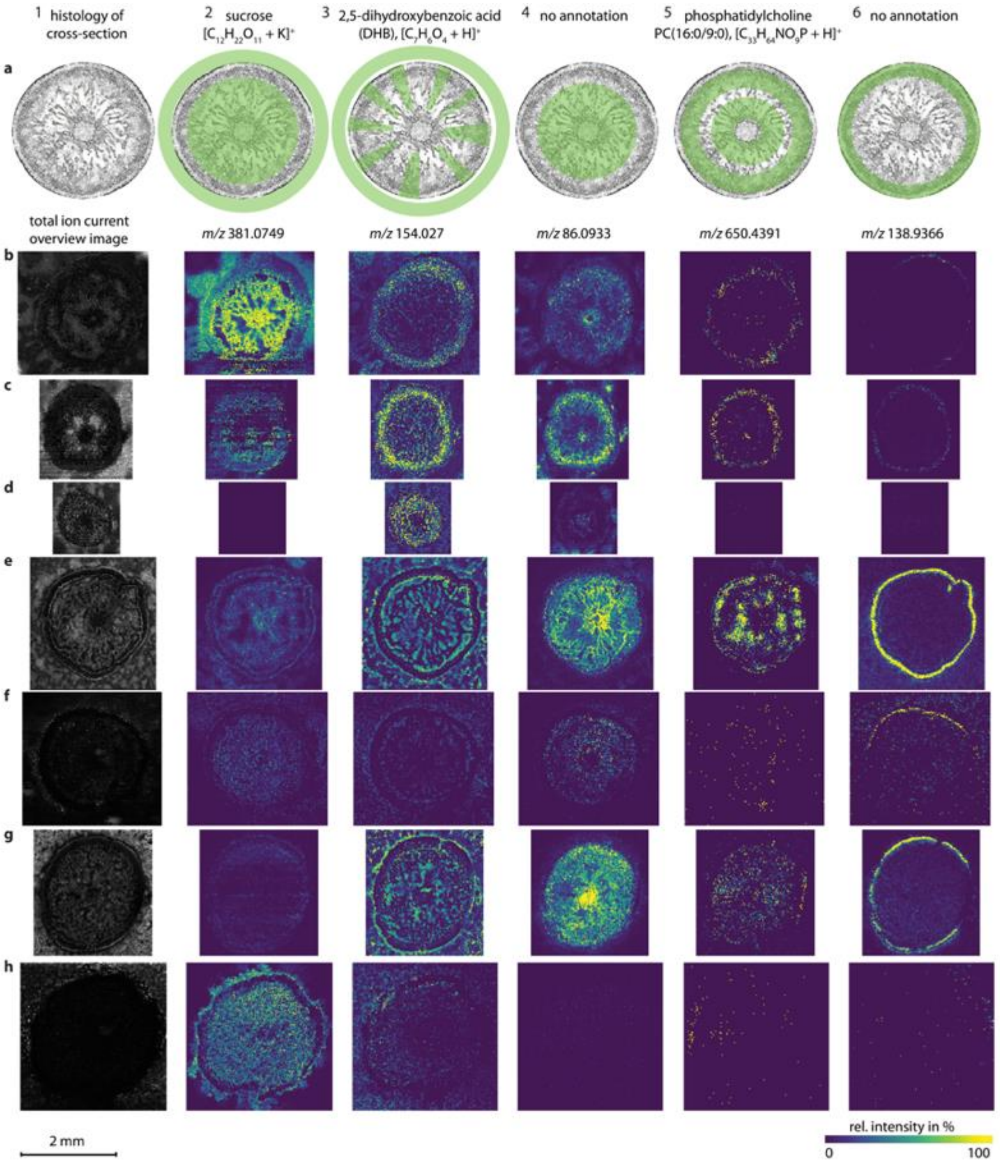

Supplementary Figure 5 | Distribution of sucrose and other small molecules across seagrass roots. Replicates and controls for matrix-assisted laser desorption/ionization mass spectrometry imaging in rows **b** to **h**, shown for five different compounds in columns 2 to 6. **a,** Column 1 shows a schematic of a cross section through a seagrass root. Column 2 to 6, row **a** show overlays of the seagrass root and the distribution patterns of different metabolites in green. Samples in rows **b**-**f,** were embedded and cryo-sectioned in carboxymethyl cellulose embedding medium. Samples in rows **g** and **h** were not embedded for cryo-sectioning to test if the sucrose leaked into the aqueous embedding medium. The sucrose at the outer boarder of the root appeared to leak into the embedding medium. Ion images in column 1 show the total ion current (TIC) image for each dataset. Metabolites in column 3 to 6 show different distributions that were consistent across the 7 control datasets. All metabolite annotations are based on putative metaspace annotations at 5 % FDR. For further descriptions of each dataset see **Table S9**.

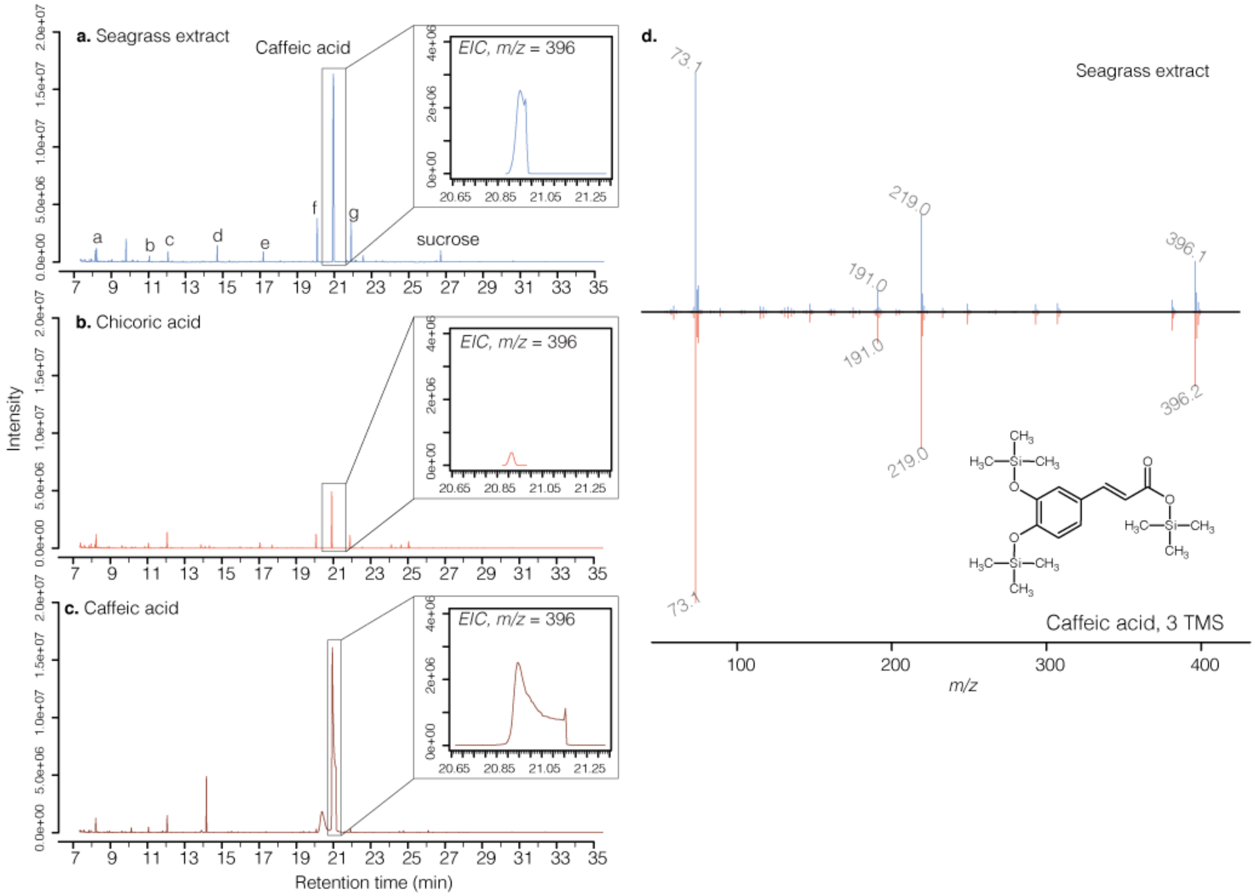

Supplementary Figure 6 | The *P. oceanica* extract contained the phenolic compounds chicoric and caffeic acid. a, GC-MS spectra of the phenolic extract used in the sediment incubation experiments. The primary peak in the extract was initially identified as caffeic acid using the NIST spectral library (**d**) and confirmed by comparison to authentic standards of **b,** chicoric acid and **c,** caffeic acid**.** Chicoric acid is a compound made up of two molecules of caffeic acid connected to one molecule of tartaric acid via ester bonds that are not stable. Because these bonds are unstable, during sample derivatization chicoric acid degrades into caffeic acid[29]. Therefore, the GC-MS cannot differentiate between the two phenolic compounds. Given that past studies using LC-MS have shown that *P. oceanica* tissues contain both chicoric acid and caffeic acid[26], we considered the caffeic acid peak from our P. oceanica extracts to represent the sum of caffeic acid and chicoric acid. **d,** The mass spectra of the caffeic acid peak in phenolic extract in comparison to the NIST database entry for caffeic acid. In **a,** lettered peaks represent a mixture of background peaks that are commonly found in our GC-MS analyses and some metabolites in trace abundances in the extract.
a = Diethyl carbamate; b = glycerol 3TMS; c = unidentified contaminant; d = proline 2TMS; e = citric acid 3TMS; f = tetradecanoic acid TMS; g= hexadecenoic acid TMS

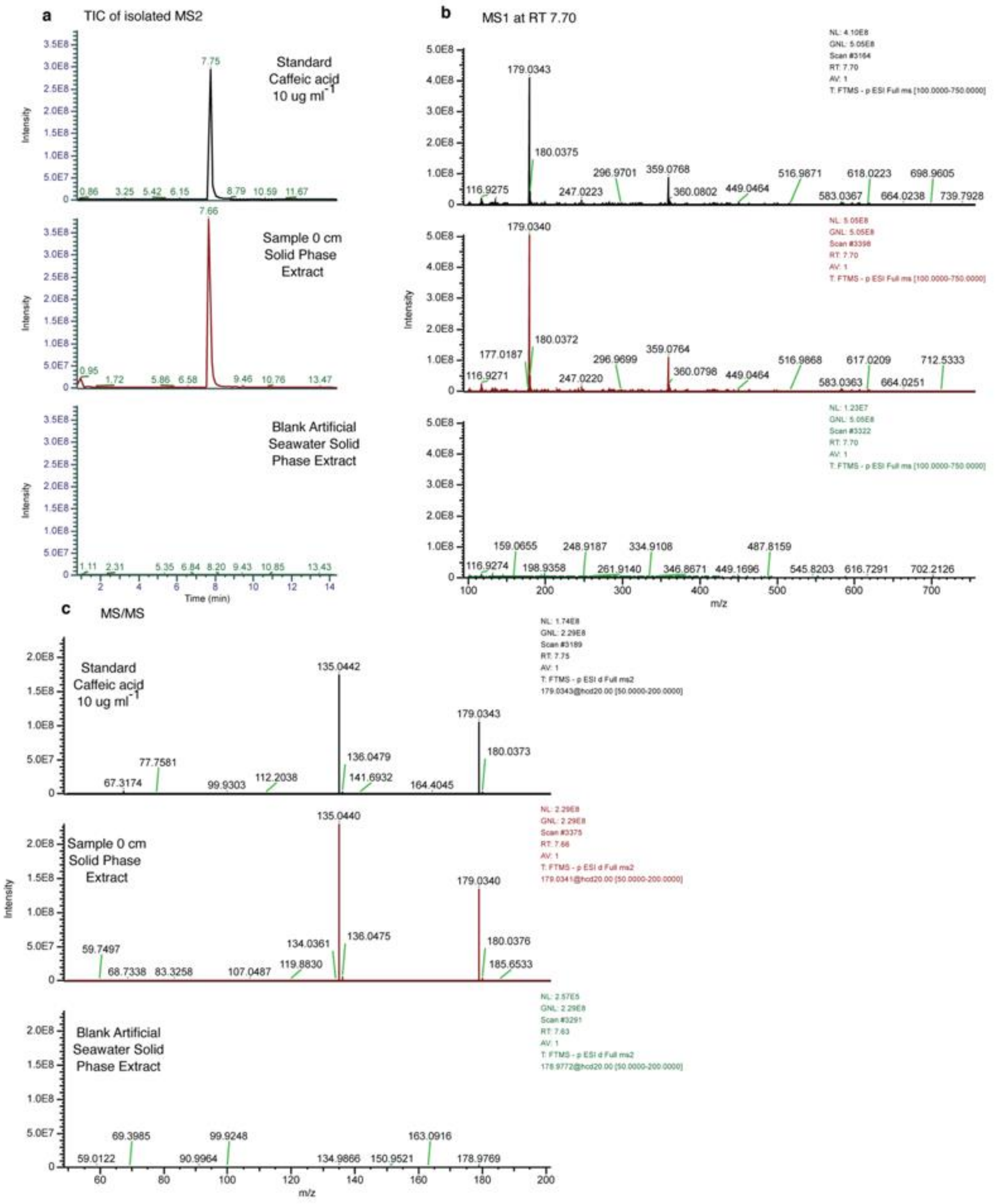

Supplementary Figure 7 | LC-MS/MS confirmation of the presence of caffeic acid in sediment pore waters underneath *P. oceanica*. a, Total ion chromatogram (TIC), **b,** MS1 and **c,** MS/MS spectra for the analytical standard for caffeic acid, a SPE-extracted sediment porewater sample, and a SPE-extracted artificial seawater blank support the correct identification of caffeic acid in the ultra-high resolution MS DOM data (**Figure 3**).

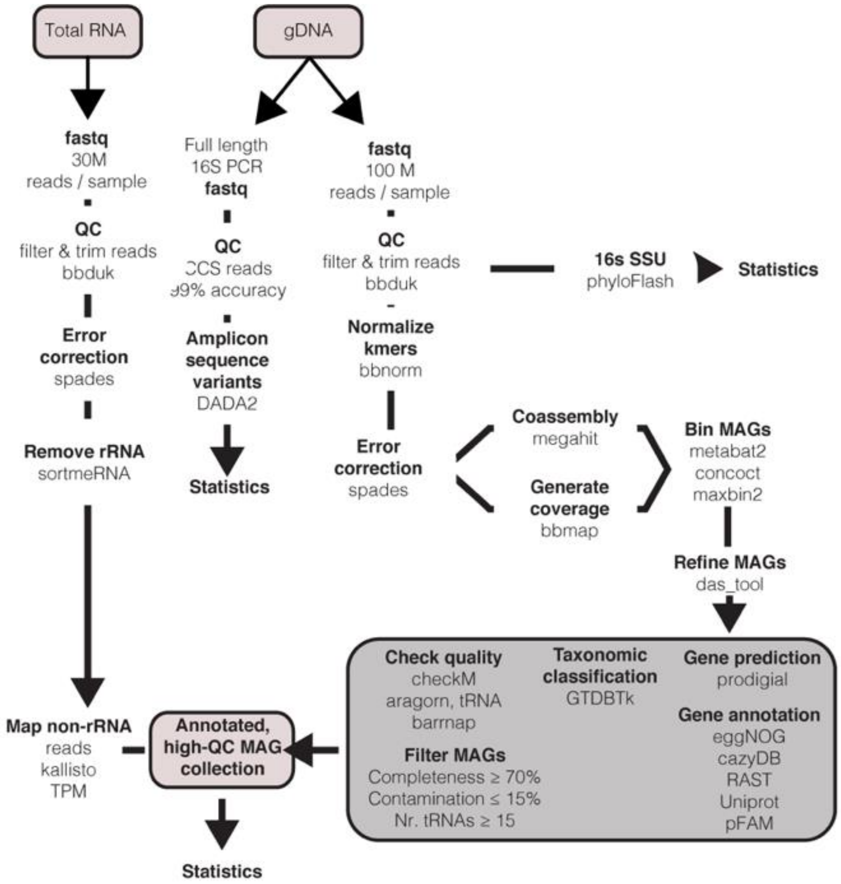

Supplementary Figure 8 | Bioinformatic pipeline used to process sediment metagenomes and metatranscriptomes. Following genomic DNA and total RNA extraction and sequencing, both full length 16S rRNA PacBio data and Illumina metagenomic libraries were used to determine the composition of the microbial community and its metabolism.

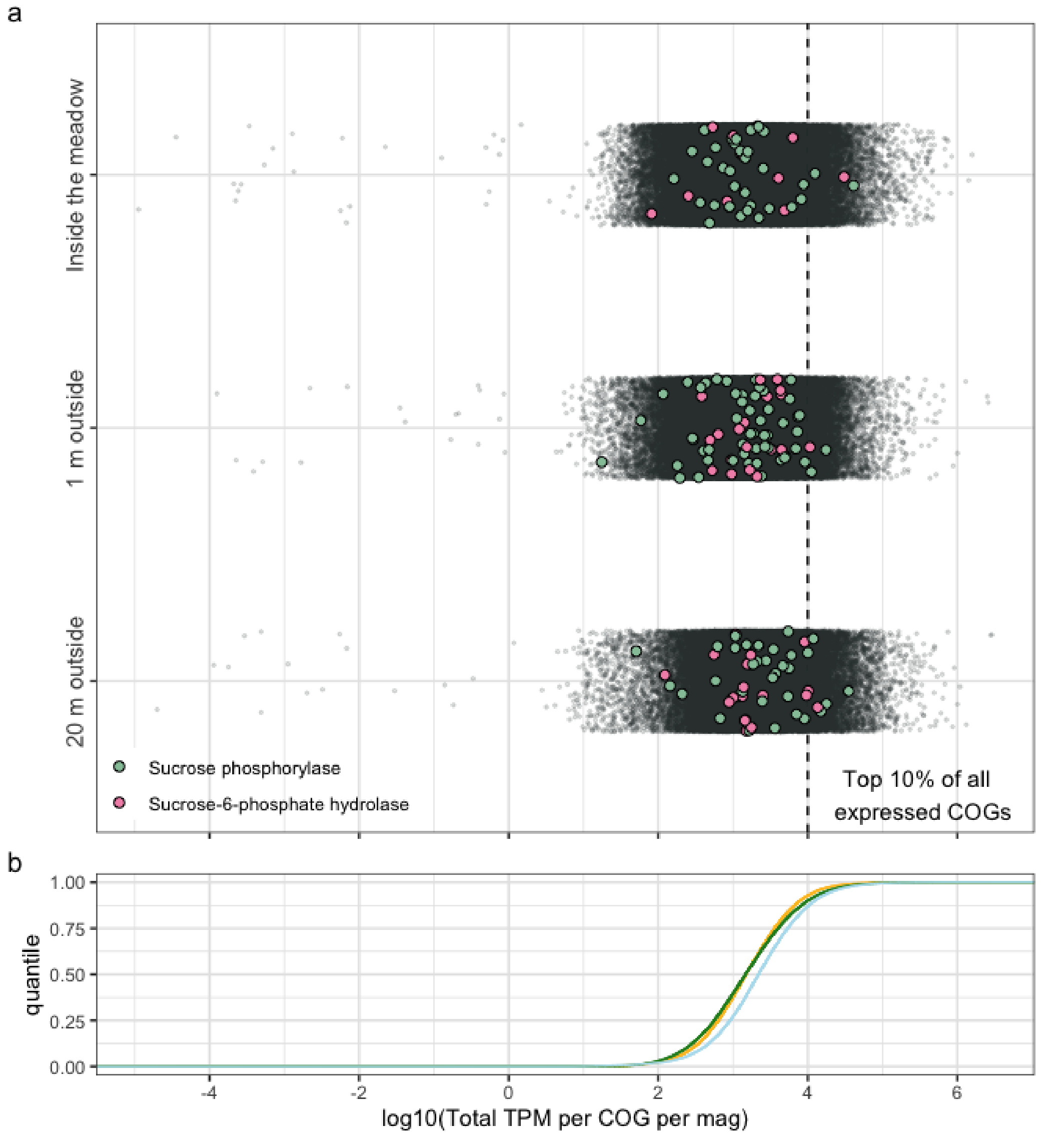

Supplementary Figure 9 | Relative expression of sucrose degrading genes was low. a, The total accumulative expression of clusters of orthologous groups of proteins per MAG from sediments collected inside, at the edge and outside a Mediterranean *P. oceanica* meadow off the island of Elba, Italy. Each point represents the total TPM of a COG category within an individual MAG summed across habitat-specific libraries. COGs predicted to degrade sucrose through either phosphorylase (green) or hydrolase activity (pink) are colored and enlarged to aid visualization. The genes within the top 10% of all expressed COGs fall to the right of the dashed black line as calculated from the cumulative distribution curves (**b**).

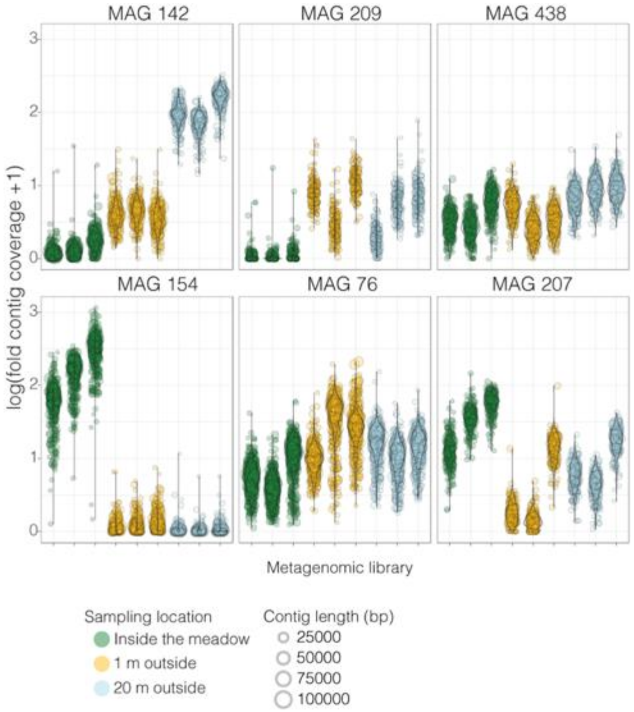

Supplementary Figure 10 | Abundance of putative sucrose specialists across sampling sites. Abundance plots of putative sucrose specialists across habitats (In, Edge, Out) based on metagenomic read coverage of individual contigs. Each point represents a contig sized by length (bp).

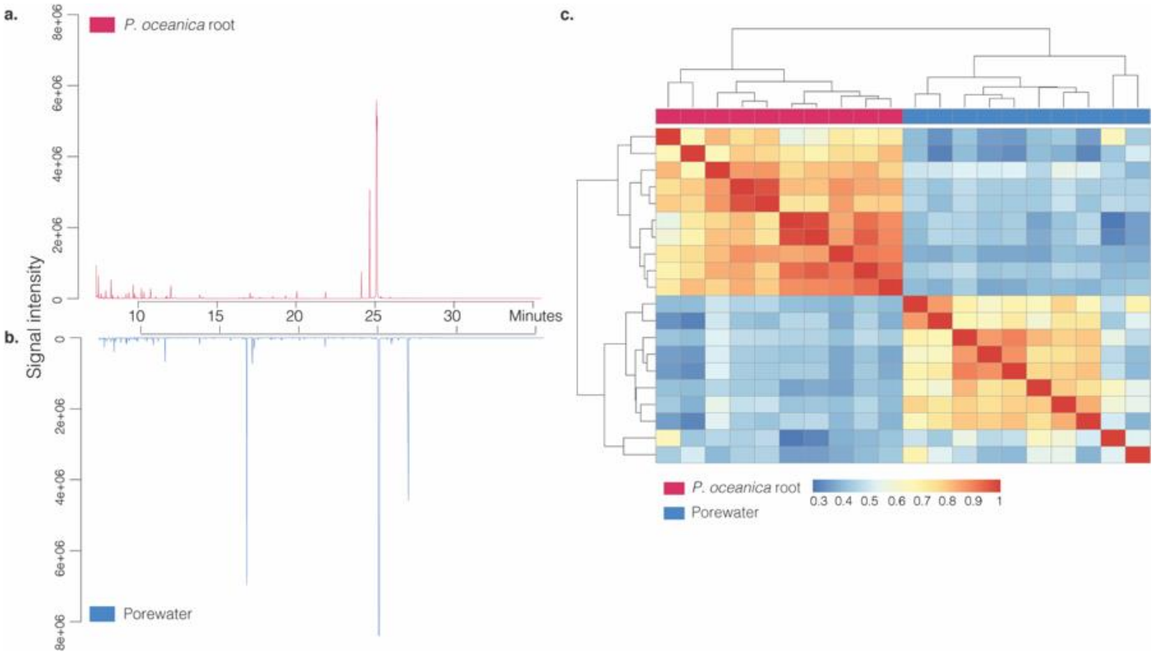

Supplementary Figure 11 | Root and porewater metabolomes were significantly different. Tail-to-tail total ion chromatograms from representative **a,** *P. oceanica* root (red) and **b,** pore water metabolomes (blue) show variation in measured compounds. **c,** supporting cluster analysis and heat map of 10 randomly selected samples for each sample type support visual assessment that *P. oceanica* roots and porewaters significantly varied in their metabolite composition. Heatmap shows the correlation between samples in terms of metabolite composition.

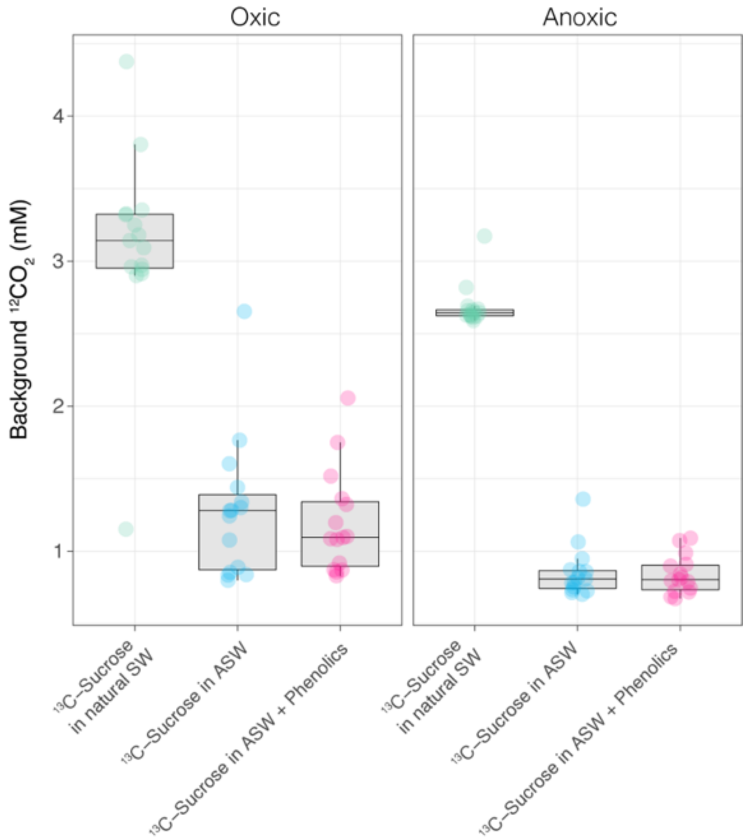

Supplementary Figure 12 | Background $^{12}CO_2$ concentrations from sediment incubations showed that the addition of phenolics isolated from seagrass tissues did not increase background microbial metabolism. Box-and-whisker plots and overlay of individual points show the distribution of background $^{12}CO_2$ levels, as a proxy for dissolved inorganic carbon concentrations (DIC), are similar between artificial seawater (ASW) experiments (blue and pink), regardless of the addition of phenolics to the incubations. Individual points represent $^{12}CO_2$ values from all sampling time points (t=0 hr, 3 hr, 6 hr, 12 hr, 24 hr) across experimental replicates (n=3 / treatment). Center line is the median, box limits represent the upper and lower quartiles, whiskers are 1.5x the interquartile ranges, outliers are not shown. These data indicate that any additional organic compounds that may have been present in the phenolic extracts, and subsequently added to the sediment incubations, did not stimulate the background metabolism of the sediment microorganisms. Background DIC levels in 13C-Sucrose incubations conducted in natural seawater (SW) where higher than in ASW treatments.
