# Supplementary material for: Sugars dominate the seagrass rhizosphere
Source: Nat Ecol Evol. 2022 May 2;6(7):866–77. doi: 10.1038/s41559-022-01740-z (PMC9262712; doi:10.1038/s41559-022-01740-z)
Supplement: Supplementary file 2 — Reporting Summary [file 41559_2022_1740_MOESM2_ESM.pdf]

nature portfolio

Corresponding author(s): Emilia Sogin, Nicole Dubilier, Manuel Liebeke

Last updated by author(s): Feb 16, 2022

Reporting Summary

Nature Portfolio wishes to improve the reproducibility of the work that we publish. This form provides structure for consistency and transparency in reporting. For further information on Nature Portfolio policies, see our Editorial Policies and the Editorial Policy Checklist.

Statistics

For all statistical analyses, confirm that the following items are present in the figure legend, table legend, main text, or Methods section.

n/a	Confirmed	
☐	☒	The exact sample size (*n*) for each experimental group/condition, given as a discrete number and unit of measurement
☐	☒	A statement on whether measurements were taken from distinct samples or whether the same sample was measured repeatedly
☐	☒	The statistical test(s) used AND whether they are one- or two-sided<br>*Only common tests should be described solely by name; describe more complex techniques in the Methods section.*
☒	☐	A description of all covariates tested
☐	☒	A description of any assumptions or corrections, such as tests of normality and adjustment for multiple comparisons
☐	☒	A full description of the statistical parameters including central tendency (e.g. means) or other basic estimates (e.g. regression coefficient) AND variation (e.g. standard deviation) or associated estimates of uncertainty (e.g. confidence intervals)
☒	☐	For null hypothesis testing, the test statistic (e.g. *F*, *t*, *r*) with confidence intervals, effect sizes, degrees of freedom and *P* value noted<br>*Give P values as exact values whenever suitable.*
☒	☐	For Bayesian analysis, information on the choice of priors and Markov chain Monte Carlo settings
☒	☐	For hierarchical and complex designs, identification of the appropriate level for tests and full reporting of outcomes
☒	☐	Estimates of effect sizes (e.g. Cohen's *d*, Pearson's *r*), indicating how they were calculated

Our web collection on statistics for biologists contains articles on many of the points above.

Software and code

Policy information about availability of computer code

Data collection	No software was used for data collection.
Data analysis	MsConvert<br>XCMS, v2.99.6<br>CAMERA, v1.32.0<br>Mass Hunter Suite<br>Scils 2019a<br>Dada2<br>phyloFlash, v2.0, https://github.com/HRGV/phyloFlash<br>phyloseq, v1.26.1<br>BBmap suite, v38.34, sourceforge.net/projects/bbmap<br>SPAdes, v3.12<br>MEGAHIT, v1.13<br>MetaBAT, v0.32.5<br>concoct, v1.0.0<br>MaxBin, v2.2.6<br>DAS Tool, v1.1.1<br>CheckM, v1.0.7<br>GTDBTk, v0.2.1<br>Prodigal, v2.6.4<br>dbCAN, v2.0,<br>diamond blastp, v0.9.25<br>interproscan, v.5.36-75

EggNOG mapper, v2.0
SortMeRNA, v2.1b
Kallisto, v0.46
GToTree, v1.4.14
HHMER3
MUSCLE
TrimAl
IQ-TREE

For manuscripts utilizing custom algorithms or software that are central to the research but not yet described in published literature, software must be made available to editors and reviewers. We strongly encourage code deposition in a community repository (e.g. GitHub). See the Nature Portfolio guidelines for submitting code & software for further information.

Data

Policy information about availability of data

All manuscripts must include a data availability statement. This statement should provide the following information, where applicable:

- Accession codes, unique identifiers, or web links for publicly available datasets
- A description of any restrictions on data availability
- For clinical datasets or third party data, please ensure that the statement adheres to our policy

Sequence data from this study were deposited in the European Nucleotide Archive under accession numbers PRJEB35096 and PRJEB40297 using the data brokerage service from the German Federation for Biological Data (GFBio), in compliance with the Minimal Information about any (X) Sequence (MIxS) standard. Metabolomics data were deposited in Metabolights (https://www.ebi.ac.uk/metabolights/) under accession numbers MTBLS1570, MTBLS1610, MTBLS1579, and MTBLS1746. All other datasets are available at zenodo.org under doi: 10.5281/zenodo.3843378.

Field-specific reporting

Please select the one below that is the best fit for your research. If you are not sure, read the appropriate sections before making your selection.

☐ Life sciences ☐ Behavioural & social sciences ☒ Ecological, evolutionary & environmental sciences

For a reference copy of the document with all sections, see nature.com/documents/nr-reporting-summary-flat.pdf

Ecological, evolutionary & environmental sciences study design

All studies must disclose on these points even when the disclosure is negative.

Study description

Porewater profiles and sediment samples were collected to describe the microbial ecology of the seagrass rhizosphere. Due to the complexity of the associated datasets, please refer to Tables S1 and S2 for sample sizes and statistical approaches used in this study unless otherwise indicated below.

Research sample

Porewater metabolomics. Porewater samples were collected at multiple locations and multiple time points across a multi-year study exploring the metabolite composition underneath seagrass meadows. Depth profiles consisted of taking 2 mL of porewater every 5 cm from the sediment surface to -30 or -40 cm beneath the meadow. In Sant'Andrea Bay, Elba, Italy, porewater samples were collected underneath the meadow, 1 m and 20 m away from the meadow. Depending on sampling location and month sampled, 6-9 individual depth profiles were collected from each sampling site. Porewater samples from Galanzana Bay, Elba, Italy, were also collected across a 24 hour period using fixed lances to reduced variation based on sampling location: At each time point we collected between 6 and 10 samples, as some samples were lost due to clogging of the porewater lance at the time of sampling.

Dissolved organic carbon. 20 mL of porewater samples were also collected for dissolved organic carbon analysis from underneath, 1 m and 20 m away from a seagrass meadow in Sant'Andrea Bay, Elba, Italy (n=6). From each site, a subset of these samples (n=3) were also analyzed for dissolved organic matter composition.

Seagrass metabolomics. Replicate seagrass samples (n=6 per sampling time point) for metabolomic analysis were collected from Galanzana Bay, Elba, Italy over a 24 hour time period at the same time as the 24 hour samples collected for porewater metabolomics. Seagrass samples were used to measure bulk sucrose concentrations from the seagrass leaves, rhizomes and root tissues, as well as explore the distribution of sucrose within the seagrass root (n=8).

Sediment samples. Sediment samples were collected from Sant'Andrea Bay Elba, Italy for metagenomic and metatranscriptomic analysis. Samples were taken underneath the seagrass meadow, 1 m and 20 m away from the seagrass meadow (n=3 per site).

Metabolic activity experiments. Sediment cores were collected underneath and 1 m away from seagrass meadow from Sant'Andrea Bay, Elba, Italy (n=3). Sediments were incubated under oxic or anoxic conditions, and in the presence or absence of phenolic compounds. 13C-sucrose consumption was monitored over time. Sucrose respiration rates were calculated from these incubations, but not statistically compared.

Sampling strategy

Porewater samples were collected using a steel lance (1 m long, 2 µm inner diameter) outfitted with a wire mesh (63 µm) to prevent the intake of sediment and seagrass, porewater was slowly extracted from sediments into sterile syringes.

Individual seagrass plants were collected by hand and immediately immersed in liquid nitrogen to halt changes in metabolite composition during sampling.

Sediment samples for metagenomic and metatranscriptomic analyses were collected using push cores. Directly after collection, cores were sectioned into 5 cm slices and frozen at -20 °C. A subsample of each sediment slice was also preserved in RNAlater (SigmaAldrich) for RNA extraction.

Dissolved organic carbon (DOC) and dissolved organic matter (DOM) samples were collected in parallel with porewater metabolomic samples from inside, at the edge and 20 m outside a P. oceanica seagrass meadow in October 2016. DOC/DOM samples were filtered through pre-combusted (500 °C, 4 h) Whatman GF/F filters (0.7 µm) into 20 mL acid-washed and pre-combusted scintillation vials. Samples were acidified to pH 2 using 25% hydrochloric acid and stored at 4 °C until analysis.

Samples for measuring the rate of sucrose consumption by seagrass sediments were frozen for metabolomic analysis at each sampling time point (0, 3, 6, 12 and 24 hours post 13C-sucrose introduction). Furthermore, samples for cavity ring-down spectroscopy to measure the production of 13CO2 were also collected at each time point by halting biological activity in 3 mL of each sample using 50 µl saturated HgCl2 solution. For each experimental condition 3 replicate incubation bottles were prepared from 3 different sampling cores inside and 1 m away from the seagrass meadow. .

For all samples collected, sample size was chosen according to sizes used in comparable studies.

Data collection	All metabolomic data was collected using either gas chromatography-mass spectrometry or mass spectrometry imaging using instruments at the Max Planck Institute for Marine Microbiology by Dr. M. Sogin, Dr. B. Geier and D. Michellod. Dissolved organic carbon and dissolved organic matter samples were analyzed by Dr. M. Seidel at the University of Oldenberg. Dr. S. Ahmerkamp collected in situ oxygen concentrations from a seagrass meadow in Sant'Andra Bay, Italy. P. Bourceau and S. Schorn collected cavity ring-down spectrometer data from sediment incubation experiments. The gene expression data were generated by DNA and RNA sequencing at the Max Planck Genome Centre in Cologne under the supervision of Dr. B. Huettel.
Timing and spatial scale	Initial metabolomics porewater profiles were collected from Sant'Andrea Bay, Elba, Italy in April, July and October 2016. Further porewater samples and seagrass tissue samples were collected across a 24 hour period from Galanzana Bay, Elba, Italy in May 2017. Porewater samples collected underneath seagrass meadows off Twin Cayes and Carrie Bow Caye, Belize were collected in April 2017, and Kiel Bight, Germany in August 2018. All porewater profiles consisted of samples taken every 5 cm from the sediment surface to 30 to 40 cm below the sediment surface. Sediment samples for metagenomics and transcriptomics were collected in October 2016 in Sant'Andrea Bay. Metabolic activity experiments using seagrass sediments from Sant'Andrea Bay were performed in September 2019 with freshly collected sediment material.
Data exclusions	No data were excluded from the analysis.
Reproducibility	All attempts to repeat the experiments were successful.
Randomization	All samples were randomly collected from seagrass habitats without a priori expectations that the sampling would influence the analysis.
Blinding	Blinding was not preformed because it was not relevant to this study. This study was an exploratory investigation into the microbial ecology of seagrass meadows, without a priori expectations that would influence the analysis.

Did the study involve field work? ☒ Yes ☐ No

Field work, collection and transport

Field conditions	Field work was conducted across a wide variety of field sites and time points during this multi-year study.
Location	Samples were collected at the following locations:<br>Sant'Andrea Bay, Elba, Italy (42° 48'29.4588" N; 10° 8' 34.4436" E ; 6-8 m water depth)<br>Galanzana Bay, Elba, Italy (42° 44'9.438" N; 10° 14' 16.3032" E; 2 m water depth)<br>Caribbean at Carrie Bow Cay, Belize (N 16° 04' 59"; W 88° 04' 55"; 2 m water depth)<br>Twin Cayes, Belize (N 16° 50' 3"; W 88° 6' 23", 2 m water depth)<br>Baltic Sea off the coast of Kiel, Germany (54° 27' 26.56256" N; 10° 11' 33.1908" E, 2 m water depth)
Access & import/export	All samples collected outside of the European Union were collected under the guidance of the Carrie Bow Cay Laboratory, Caribbean Coral Reef Ecosystem Program, National Museum of Natural History, Washington DC. These samples and samples collected from waters within the European Union were collected in compliance with local, national and international laws.
Disturbance	No disturbance was caused by the study.

Reporting for specific materials, systems and methods

We require information from authors about some types of materials, experimental systems and methods used in many studies. Here, indicate whether each material, system or method listed is relevant to your study. If you are not sure if a list item applies to your research, read the appropriate section before selecting a response.

Materials & experimental systems

n/a	Involved in the study
☒	☐ Antibodies
☒	☐ Eukaryotic cell lines
☒	☐ Palaeontology and archaeology
☒	☐ Animals and other organisms
☒	☐ Human research participants
☒	☐ Clinical data
☒	☐ Dual use research of concern

Methods

n/a	Involved in the study
☒	☐ ChIP-seq
☒	☐ Flow cytometry
☒	☐ MRI-based neuroimaging
